# The old-style public health measures and the novel coronavirus outbreak

**DOI:** 10.1590/S1677-5538.IBJU.2020.0282

**Published:** 2020-07-31

**Authors:** Antonio Cassio Assis Pellizzon

**Affiliations:** 1 Centro de Câncer AC Camargo Departamento de Oncologia Radiológica São Paulo SP Brasil Departamento de Oncologia Radiológica, Centro de Câncer AC Camargo, São Paulo, SP, Brasil

*To the editor,*

Quarantines and travel bans have been the first response of Public Health against new infectious diseases. In health practice the quarantine refers to the separation of persons or communities who are exposed to an infectious disease. Isolation, in contrast, applies to the “separation” of persons who are known to be infected. Of importance is that isolation and quarantine can be imposed by law or voluntary. However, these classical public health measures are usually of limited utility for highly transmissible diseases. Other tools that Public Health have at hand are social distancing and community containment. The primary goal of such measures is to prevent person-to-person spread of disease, trying to interrupt transmission.

China has been preparing to contain future pandemics by applying lessons learnt from the SARS outbreak in 2003 (1). But public health measures taken at that time were successful for SARS because the vast majority of contaminated patients were symptomatic, thus identifiable and could be isolated. Delays in detection of infected patients may be related to subclinical symptoms and diverse initial manifestations in this new pandemy. Better assessments of viral shedding are needed to our understanding of the transmission dynamic and infection-control practices.

Early detection of Covid-19 is difficult because of its apparent subclinical nature in some persons (2). Although asymptomatic transmission has been suggested, it is uncertain if or when patients infected with SARS-CoV-2 initiate transmissions. Early data suggest that SARS-CoV-2 infection has higher estimated reproductive number (2.2 vs. 0.9) and a shorter estimated serial interval distribution (7.5 days vs. 12.6 days) when compared to MERS-CoV infection that occurred in 2015 (3, 4).

Whether these rigorous measures will result in victory depends on many factors (1): What is the proportion of subclinical disease that will never turn to symptomatic or mildly symptomatic, hence not be identified and isolated? (2). For infected persons what day is the peak viral shedding? (3). Does viral shedding occur before onset of symptoms? (4). Is there any other form of viral shedding?

The answers to all these and other questions will certainly drive the needed responses.
